# Collaborative Swarm Robotics for Object Transport via Caging

**DOI:** 10.3390/s25165063

**Published:** 2025-08-14

**Authors:** Nadia Nedjah, Karen da Silva Cardoso, Luiza de Macedo Mourelle

**Affiliations:** 1 Department of Electronics Engineering and Telecommunications, State University of Rio de Janeiro, Rua são Francisco Xavier 524, Rio de Janeiro 20000-000, Brazil; cardosokaren.ck@gmail.com; 2 Department of Systems Engineering and Computation, State University of Rio de Janeiro, Rua são Francisco Xavier 524, Rio de Janeiro 20000-000, Brazil; ldmm@eng.uerj.br

**Keywords:** cooperative transport, swarm robotics, object caging

## Abstract

In swarm robotics, collective transport refers to the cooperative movement of a large object by multiple small robots, each with limited individual capabilities such as sensing, mobility, and communication. When working together, however, these simple agents can achieve complex tasks. This study explores a collective transport method based on the caging approach, which involves surrounding the object in a way that restricts its movement while still allowing limited motion, effectively preventing escape from the robot formation. The proposed approach is structured into four main phases: locating the object, recruiting additional robots, forming an initial cage around the object, and finally, performing the transportation. The method is tested using simulations in the CoppeliaSim environment, employing a team of Khepera-III robots. Performance metrics include execution time for the search and recruitment phases, and both execution time and trajectory accuracy, via a normalized error, for the transport phase. To further validate the method, a comparison is made between the caging-based strategy and a traditional pushing strategy.

## 1. Introduction

Collective transport in swarm robotics is defined as a group of robots used to transport a specific object, with dimensions significantly larger than those of a single robot, from one point to another on an arena. The robotic swarm is characterized by a set of robots that, through behaviors similar to those of insects or animals exhibiting collective intelligence, perform a given task [1,2]. The group of robots that make up the swarm generally share the same architecture and are robots that individually perform simple functions such as sensing, locomotion, and basic communication. However, cooperatively, they are capable of performing complex functions such as transport. Collective transport has various applications and consequently numerous studies are based on it. Three transport strategies stand out: pushing, grasping, and caging [2,3,4,5].

In this work, caging is used as the transport strategy. This method is characterized by the complete enclosure of an object’s perimeter by a swarm of robots for a specific objective. The main advantage of caging in transporting an object is the coordinated and cohesive progression. In this method, the forces applied to the object during transport, from all directions, prevent any significant deviation of the object from its programmed path. When completely enclosed, the forces applied by each robot to the object complement each other, allowing for straight-line movement. In other words, while the robots positioned at the rear of the object apply forces that generate movement, the robots positioned at the sides and front of the object exert forces that keep it caged and moving in the desired direction. There are numerous applications of collective transport through caging. Among these are the transport of fragile objects; the assembly of structures, the manipulation of objects at the nanoscale and microscale; logistical optimization in confined spaces; and medical applications, involving the controlled transport of medications and medical devices.

The problem of collective transport encompasses fundamental processes beyond the transport itself. These include the following: the swarm of robots searching for the object in the arena; once the object is detected, recruiting the swarm to the detection region; the initial positioning of the robots around the object’s perimeter, forming an enclosure structure; and finally, the transport itself. Therefore, this study is divided into four distinct stages: the first dedicated to the swarm’s search for the object in the arena; the second to the recruitment of the swarm to the region where the object is detected; the third focused on the initial positioning of the robots around the object, creating the formation; and lastly, the fourth stage, dedicated to the transport itself.

In this work, a method for performing the collective transport of cylindrical objects through caging is proposed. This method is based on the distributed coordinated concept, which is characterized by extensive communication among the robots, enabling them to organize as a swarm and complete all stages of collective object transport. This is achieved through direct information obtained via the individual sensors of each robot, or indirectly through message exchanges between the swarm members. This methodology offers a more precise transport trajectory when compared with the simple object-pushing strategy.

Mainly, the contributions of this work center around addressing the key challenges associated with each stage of collective transport by caging. First, the work introduces a simplified, random-search strategy for object detection, allowing the focus to remain on the development of an effective caging mechanism. This decision lays the groundwork for integrating more advanced search strategies, such as ant colony optimization, in future work. In the recruitment stage, the work proposes a decentralized mechanism in which the first robot to detect the object becomes the recruiter and sequentially recruits other swarm members through direct signaling. This process is enhanced by the use of Braitenberg-inspired behavior to guide robots safely around the object, minimizing collisions and facilitating precise positioning. For the transport stage, the methodology defines a control strategy that maintains the caging formation by assigning functional roles to robots based on their positions relative to the object. Rear robots primarily generate propulsion, while side and front robots contribute to both motion and containment, ensuring that the object remains enclosed and follows the desired trajectory. Additionally, the work emphasizes the critical role of synchronized inter-robot communication during transport. Communication ensures that all robots align their actions before initiating movement and at each waypoint, maintaining formation integrity throughout the task. Together, these contributions form a coherent and adaptable framework for caging-based object transport in swarm robotics.

The remainder of this paper is organized into six sections. Initially, in Section 2, we introduce the concept of collective transport and describe its main strategies. Additionally, we formally define the problem, focusing on the collective transport strategy of caging, which is the approach adopted in this work, and address the main challenges. Then, in Section 3, we review relevant works. We identify the utilized distinct approaches: either distributed or coordinated. We then establish the distributed coordinated approach adopted in this research. After that, in Section 4, we introduce the models applied to the object caging process. This includes the presentation of the mathematical model used for object caging, as well as the strategies employed for the movement of the robots and for maintaining the caging during transport. This strategic approach is based on the use of state machines and PID controllers, which are detailed and discussed in this section. Then, in Section 5, we sketch the algorithms developed for the proposed approach implementation, structured according to the process stages. After that, in Section 6, we discuss implementation and simulation issues and we give the required simulation parameters. Next, in Section 7, we focus on presenting the results obtained for the collective transport method using caging. Initially, we discuss the results achieved in the search and recruitment stages. Then, the results related to the transport stage are presented. Additionally, we compare the performance of the work performed here to an existing study. Subsequently, in Section 8, we conclude by providing an analysis of the main conclusions that one can draw from the implementation of the proposed collective transport by caging. Additionally, we suggest some possible directions for future work.

## 2. Object Collective Transport Problem

For collective transport using a swarm of robots, there are three main strategies that stand out: pushing the object, grasping it, or caging it.

The pushing strategy consists of positioning robots at the rear of the object to be transported, so that the force applied by these robots generates the movement of the object in the desired direction. In this strategy, at each step, the robots reposition themselves to ensure that the object is pushed in the desired direction with the least possible deviation. Since with each push the object may deviate from the intended orientation to some degree, it is necessary after each push to check whether the orientation has been maintained or if the robots need to reposition around the object to ensure that the resultant force generated by the swarm in the next push corrects the direction of transport [6]. Maintaining direction and reducing deviations are the main challenges of this strategy, in addition to those related to swarm transport, such as communication between the robots.

The grasping strategy is based on a swarm of robots equipped with physical mechanisms that enable them to latch onto the object, thereby holding and transporting it by pulling, or even lifting it [7]. Compared to the pushing strategy, the impact of trajectory deviation during transport is significantly lower in this strategy since the robots are physically connected to the object, and there is no need for repositioning at each step. In addition to requiring a swarm of robots with specific physical characteristics for implementing this strategy, another significant challenge is analyzing and calculating the optimal arrangement of the swarm along the object to ensure transport efficiency.

Caging can be defined as the act or effect of enclosing or imprisonment in a cage. In the study of swarm robotics, caging involves enclosing the perimeter of an object by a swarm of robots for a specific purpose, with the most popular objective being transport [2]. The main advantage of the caging strategy in transporting an object, compared to the pushing strategy, is the coordinated and cohesive progression of the transport. In this strategy, the forces applied to the object during transport, from all directions, prevent any significant deviation of the object in unintended directions from occurring. When fully enclosed, the forces applied by each robot to the object complement each other, enabling straight-line movement. So, while the robots positioned at the rear of the object apply forces that generate movement, the robots positioned on the sides and front of the object exert forces that keep it caged and moving in the desired direction. Although caging transport is efficient and applicable to various purposes, its major disadvantage lies in the challenges during implementation. Multiple parameters must be evaluated at each step. Continuous analysis of parameters and variables related to caging is required.

### 2.1. Problem Definition

Collective transport by caging is based on transporting an object from one position on an arena to another through the collective work of multiple robots using the caging strategy. In general, the problem of collective transport encompasses other complementary processes. Therefore, for the cohesive development of this work, it has been subdivided into three stages: object search, recruitment, and transport. For each of these stages, there are various parameters and variables formally defined in this section.

The arena is the setting where the execution for all stages of the work is conducted. Although the search stage is defined as the first stage to be implemented, the discretization of the arena precedes this stage. This is because, for the search to begin, the manner in which the robots will traverse the arena and the elements that will compose it must be established beforehand to facilitate the object search process. So, in order to make the search stage efficient, waypoints, also called reference points, are placed in the arena. These reference points are fixed throughout the arena, with $L=\{\lambda_{0},\lambda_{1},\ldots ,\lambda_{\Lambda -1}\}$ being the set of Λ landmarks. For each reference point $\lambda_{i}$, a neighborhood is defined through the function $\eta_{\left(\lambda_{i}\right)}$, which can be set as required by the application. The main function of the reference points is to enable the widest possible dispersion of the robots throughout the arena, as well as to allow the robot to identify which regions it has already examined.

Let R={0,1,…,ρ−1} be the set of ρ robots forming the swarm. The robots are characterized by the position of their center of mass $\left(x_{i},y_{i}\right)$ and the bearing $\beta_{i}$. Variable $p_{i}$ represents the current position and orientation information of robot *i*, $p_{i}={\left[x_{i},y_{i},\beta_{i}\right]}^{T}$. Let *w* be the width of the robot. The first robot to detect the object is called the recruiter and denoted by $r^{*}\in R$. Furthermore, the recruitment order of the robots, which will be carried out by robot $r^{*}=r_{i}$, is determined according to the definition given by function $\mu_{i}$. Each robot *i* has a total of *s* sensors, and these sensors are structured as follows: sensors〈detect,id〉, where detect is a Boolean value (1 when an item is detected and 0 if not) and id represents the identifier number of the detected item.

Caging involves an important factor that significantly contributes to the complexity of the problem. It is related to the initial positioning of the robots around the object to be enclosed. Various factors influence the efficient arrangement of the robots along the perimeter of the object, such as the shape of the object, the number of available robots, and the physical characteristics of both the robots and the object.

The object to be transported has a cylindrical shape, a choice made to minimize the complexity associated with irregularly shaped objects. Notably, no dynamic model is defined for the object; instead, the mathematical model is specifically developed for cylindrical shapes. Furthermore, any irregularly shaped object can, in principle, be enclosed within a cylindrical container. However, for non-cylindrical objects, a careful selection of the robots’ initial positions in the enclosure formation is necessary. As future work, we plan to extend the proposed model to accommodate other object shapes, which will require modifications to some equations to account for different geometries.

The required number of robots ρ for caging the object is determined according to the mass *M* of the object and that of the used robots and the dimensions of the chosen object, where $r_{⊕}$ is the radius of the object and $C_{⊕}=\left(x_{⊕},y_{⊕}\right)$ is the position of its center of mass in the arena. Note that this position is unknown to the robots of the swarm. Furthermore, let $T_{r^{*}}$ be the sequence of reference points traversed by the recruiter robot $r^{*}$ from its starting point in the arena to the object detection point. This sequence is traversed in reverse by the object during the transport stage. It is noteworthy to emphasize that this work is focused more on providing a solid strategy for the collective transport stage and not on path optimization; neither during the object search nor during its transport. Optimizing paths would require a separate study, wherein path optimization techniques could be explored and compared. For example, one can notice that there could be paths that are shorter than the one used by the recruiter during its search for the object. To keep the focus on the main objective of the research, we simply use a random search until one of the robots finds the object, and we use the same path traversed by this robot to transport the object back to the recruiter’s starting position.

Communication between the robots is carried out through messages, structured as 〈type,origin,destination,payload〉, wherein the first item contains the type of the message, the second indicates the robot identifier that issued the message, the third the robot identifier to which the message is sent, and the fourth contains the content of the message, if any. There are four types of messages depending on the situation; these are described later.

### 2.2. Main Challenges

The challenges related to the problem of collective transport by caging are associated with each stage of this work’s development.

The main challenge in the search stage is to ensure the efficiency and speed with which the swarm finds the object. Various implementation strategies can be applied at this stage, such as the use of the path construction meta-heuristic based on ants’ behavior during foraging for food [8]. In this work, we use a simple random search strategy, as we are focusing on contributing towards an efficient caging strategy instead. More efficient search strategies will be explored in future improvement of this work.

The challenges related to recruitment are associated with communication between the robots, which is extensive at this stage. The first robot to identify the object’s position becomes the swarm recruiter and is responsible for recruiting the other robots in the swarm one by one. This process is based on signal exchanges between the recruiter and each robot in the swarm, which demands significant computational cost. Another important aspect of this stage is the movement of robots around the object. They must maneuver without causing collisions with the object or other robots already in position. The concept of the Braitenberg vehicle [9,10] is applied during the recruitment. The recruitment stage also significantly depends on the initial positioning of each robot.

The challenges related to transport make the implementation complex. They involve the analysis, calculation, and maintenance of parameters associated with the robots’ movement as they cage-off the object at each step. These parameters are crucial for maintaining the formation of the robots in a caging configuration throughout the transport. The parameters aim to ensure that the robots positioned at the rear of the object exert enough force to generate movement, while the robots on the sides and front of the object exert forces related to both movement and containment, maintaining the caging and the desired direction of transport. Communication significantly impacts this implementation, as well as the requirement for the robots to communicate and synchronize their actions upon reaching and when departing from each landmark during the transport stage. Communication ensures that the transport stage is carried out synchronously, maintaining the caging of the object throughout the entire path, thus enabling the transport of the object via the prescribed trajectory. Therefore, communication between the robots is fundamental in this implementation. Once caging is completed, the transport stage begins, directing all robots towards the last reference point on the constructed path during the object search. Transport starts only when the robots are aware that the entire swarm is well-oriented. The same process occurs when reaching each reference point. Once all robots arrive at the desired point, a new orientation and transport process begins towards the next point in that path.

## 3. Related Works

There are different methods of collective transport by caging in swarm robotics. Based on the analysis of recent relevant works, we identify three main approaches: distributed, coordinated, and machine learning-based.

### 3.1. Distributed Swarm Transport

A distributed swarm transport method relies on communication among robots, allowing them to identify the object’s position and organize into a cage around it.

The work proposed in [2] uses a distributed method based on the definition of a seed robot. A robot is designated as such because it is the first to make direct contact with the object. Based on the seed robot and its position, the other robots are allocated around the object at a predefined inter-robot distance. The collective behavior of the swarm is managed by a high-level state machine, enabling the robots to participate in sequential rounds of task allocation. This allows them to position themselves around the object from distributed locations following the seed robot’s contact with the object, while maintaining the desired inter-robot distance during positioning.

The approach proposed in [4] uses the method of distributed coordinated transport to cage and transport large convex objects using a significant number of robots, which are considerably smaller than the object to be transported. Similar to the work presented in [2], the path to be followed by the caged object is translated into a sequence of intermediate points that the robots must follow to reach the final target position. The robots used in this implementation can detect the target position via their sensors. Similar to in [2], a high-level state machine governs the individual behavior of each robot.

The work presented in [11] proposes a caging strategy for a group of robots needed to transport an L-shaped object and a circular-shaped object, following a reference trajectory. In this study, robots locally estimate the closure of the object based on direct communication regarding their position relative to the object. Controlled by an architecture with different behaviors, the robots switch from approach behavior to surrounding behavior when close to the object. In surrounding behavior, robots distribute themselves around the object to form the potential cage. The robots communicate their states to neighbors until a quorum is reached.

### 3.2. Coordinated Swarm Transport

The leader-based caging transport method involves a system where a leader (either a robot or a human) controls the transport of an object by coordinating a swarm of robots. Unlike distributed transport, where robots communicate with each other, the leader-based method only requires communication between each robot and the leader.

The work presented in [12] proposes a caging formation where robots are arranged on both sides of an object to trap it while allowing limited movement. The goal is to surround and contain the object, not to create a deterministic formation. Once the caging is in place, the robots apply force perpendicular to the object’s surface to move it. To change the direction of movement, the robots must reorganize the formation. The leader, operated remotely by a human, controls the robots’ movement and the transport path as they follow the leader.

The work proposed in [13] explores a leader-based transport method in large-scale human–swarm interactions, where a human acts as the leader. It tests three system architectures: the first involves manipulating two objects to form a single one; the second extends this by transporting the unified object to a target; the third adds obstacles to the task. In these experiments, users control the swarm via keyboard commands, directing all robots to move together. The system is tested both through simulations and hardware, comparing the results to identify similarities and differences. During the experiments, the position, orientation, and task completion time of the object are tracked to analyze the leader’s strategy and trajectory.

Another coordinated transport method is presented in [14]. The authors propose a caging strategy where the control includes two phases. The first is the initialization process, which finds the satisfactory number of robots and the initial formation of robots to cage the object. The second is the movement process, which uses a simple virtual leader controller or employs translational robustness to actively choose support robots.

In [15], a control strategy based on variable internal forces is introduced for coordinating a team of three omnidirectional robots tasked with moving a cube-shaped object. Each robot has a cuboid form, resulting in contact along line segments rather than single points. The system designates one robot as the leader, responsible for driving the object forward, while the other two act as followers that apply lateral forces to maintain their fixed relative positions and orientations with respect to the object. Coordination among the robots does not rely on explicit communication; instead, it is achieved by observing the net force exerted on the object and tracking its motion. This method of implicit coordination, through shared physical interaction with the object, allows the follower robots to remain in formation and assist in transporting the object along a path known only to the leader.

### 3.3. Advancement in Swarm Transport

Research has demonstrated that reinforcement learning algorithms, such as the Double Deep Q-Network (DDQN), can be applied to the decentralized control of multi-robot formations. This approach enables each robot to autonomously learn control policies that facilitate the formation and maintenance of desired structures, thereby eliminating the need for centralized and complex mathematical models [16]. This capability is particularly relevant to swarm caging applications, in which the flexibility and scalability of the system are critical factors. The study presented in [17] is another recent work that applies machine learning to allow the control of a swarm of surface vehicles for formation tracking and optimal control. It combines a high-level displacement-based controller with a low-level reinforcement learning strategy for individual vehicles.

Furthermore, distributed reinforcement learning techniques have been employed in the control of unmanned aerial vehicle swarms, enabling agents to explore unknown areas cooperatively. These agents are capable of adapting to environmental changes while maintaining formation cohesion, even in the presence of obstacles [18]. Such adaptability is essential in swarm caging scenarios where the swarm must form containment structures around moving targets in unstructured environments.

Another significant advancement is the application of Extended Particle Swarm Optimization, which addresses practical constraints, such as limited field of view, local sensing, and restricted communication among agents. This algorithm dynamically adjusts particle weights based on the degree of aggregation and the evolution speed of the swarm, allowing efficient updates to robot velocities and directions even under kinematic limitations [19]. When applied to swarm caging-based collective transport, it contributes to the dynamic reorganization of the swarm during the caging formation process. Reinforcement learning-based methods have been employed to coordinate such systems, allowing robots with varying dynamic models to learn effective control policies for achieving and maintaining containment formations, even under uncertainty and constraints [20].

## 4. Proposed Model for Object Transport

This section presents the building blocks of the modeling used for the formation of the caging robots around the object to be transported. Section 4.1 introduces the model developed to determine the maximum and minimum number of robots required for caging and transporting the object, as well as to determine the minimum angle between the robots to guarantee the proper caging. Section 4.2 presents the design of the required controllers. Section 4.3 presents the modeling developed for the movement of the robots during the different stages of the process.

### 4.1. Swarm Size

**Figure 1:**
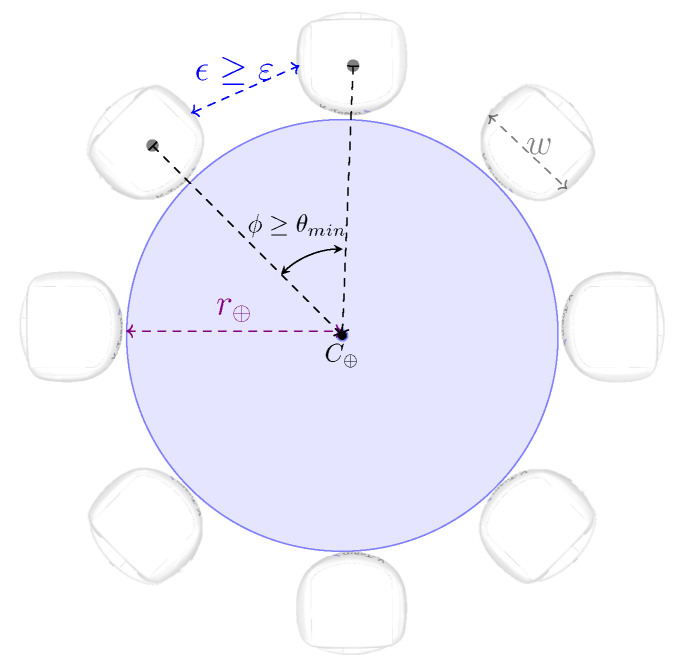
Illustration of object radius: $r_{⊕}$; minimal inter-robot distance: ε; inter-robot distance: ϵ; robot width: *w*; and minimal angle between robots’ positions while caging: $\theta_{min}$.

The caging of the robots around the object is planned according to the dimensions and characteristics of the object and the swarm. First of all, we need to identify these characteristics and define the swarm size, i.e., the minimum number of robots ρ required to transport the object, as well as determine the minimum angle that must maintained between the robots to prevent collisions. Based on the force equilibrium model, where no torque and no friction are taken into account [21], the definition of the minimum number of robots required for transporting the object is expressed in Equation (1):

(1) $$\rho_{min}=\frac{M\times A}{m\times a},$$

where *M* denotes the mass and *A* represents the acceleration of the object. The constants *m* and *a* represent the mass and acceleration of the robot, respectively. The calculation of the minimum angle between robots allows for determining the maximum number of robots that should be involved in the caging. This angle can be expressed as in Equation (2):

(2) $$\arctan\left(\frac{\theta_{min}}{2}\right)=\frac{\left(w/2+\varepsilon /2\right)}{\sqrt{{\left(w/2+r_{⊕}\right)}^{2}-{\left(w/2+\varepsilon /2\right)}^{2}}},$$

where $\theta_{min}$ represents the minimum angle between robots in the caging formation, *w* denotes the width of the robot, the constant $r_{⊕}$ represents the radius of the object, and ε is a predefined constant that translates to the minimum inter-robot space to be maintained between adjacent robots in the caging formation, defined with the goal of preventing the robots from colliding or interfering with each other during their movement, especially during re-orientation, which is performed at each repositioning to maintain the caging formation. Clearly, the positioning of the robots around the object in the caging formation is determined based on the angle ϕ, obtained by dividing the cylinder’s circular perimeter by the total number of robots ρ. These defined constants and variables are illustrated in Figure 1, wherein the angle ϕ is shown as greater than or equal to $\theta_{min}$. The angle between robots ϕ must be greater than or equal to the minimum angle $\theta_{min}$ obtained from Equation (2). The constant ϵ represents the inter-robot distance between robots, with ϵ≥ε as ε is defined considering the minimum angle $\theta_{min}$ while ϵ is for angle $\phi \geq \theta_{min}$, as illustrated in Figure 1.

### 4.2. Proportional–Integral–Derivative Controllers

PID control is chosen as the control method for the movement of the robots in the simulation environment. This choice was made due to the high reliability and efficiency of this method, as well as its widespread use in various applications that depend on continuous control.

A discrete implementation of the process controlled by the PID is sketched in Algorithm 1. Moreover, a solution is included to address potential issues of integral action wind-up [22]. The phenomenon of wind-up in the integral action of PID controllers is characterized by the delayed response of the system when the actuator operates under saturation conditions. This effect was mitigated through the implementation of two specific techniques: Back-calculation/tracking and conditional integration. The first method aims to adjust the integral term in response to actuator saturation. Therefore, when the actuator output reaches its saturation limits, the integral term is recalculated to maintain its value within the saturation limits. The conditional integration method consists of suspending the integral action under specific conditions, such as when the system error exceeds a predefined threshold or when saturation of the manipulated variable occurs. The suspension of the integral action prevents excessive accumulation of the integral term, which is the primary cause of wind-up. The implementation of these methods for mitigating integral action wind-up can be observed in Algorithm 1, wherein the conditional instructions implement the back-calculation/tracking and conditional integration techniques, respectively. Note that the thresholds $e_{b}$ and $u_{s}at$ are empirically set up.
**Algorithm 1** PID($k_{p},k_{i},k_{d},t_{s},u_{sat},e_{b},r,y$)**Ensure:** *u*;
    $e_{old}:=e$; e:=(r−y);
    $acc:=acc+\frac{\left(e+e_{old}\right)}{2\times t_{s}}$; $u:=k_{p}\times e+k_{i}\times acc+k_{d}\frac{e-e_{old}}{t_{s}}$;
    **if** 
$u>u_{sat}$
 **then**
        $acc:=acc-\left(u-u_{sat}\right)k_{i}$; $u:=u_{sat}$;
    **end if**
    **if**  $u<u_{sat}$∧ $e<e_{b}$ **then**
        $acc:=acc+\frac{\left(e+e_{old}\right)}{2\times t_{s}}$; $u=k_{p}\times e+ki\times acc+k_{d}\frac{\left(e-e_{old}\right)}{t_{s}}$;
    **end if**


PID controllers are chosen to supervise the movement of the robots and the formation of the caging because it can analyze and correct the error at each step. Therefore, during the movement, two PID controllers are used: one considering the process variable $D\left(p,p^{\prime }\right)=D\left(p_{i},\lambda \right)$, and the other $\alpha_{p^{\prime }}=\alpha_{\lambda }$. These controllers allow for the analysis and correction of the error in the robots’ movement by controlling the distance between the robot and the target position and orientation. The distance $D(p_{i},\lambda )$ is the distance between the current position of the robot’s center of mass and the aimed for landmark λ. In this work, the distance between two points is computed using the Euclidean distance, described in Equation (3):

(3) $$D\left(p,p^{\prime }\right)=\sqrt{{\left(x-x^{\prime }\right)}^{2}+{\left(y-y^{\prime }\right)}^{2}}.$$

Moreover, the angle $\alpha_{p^{\prime }}=\alpha_{\lambda }$ is formed by the robot’s bearing $\beta_{i}$ and the orientation towards the desired reference point λ. This angle is computed using Equation (4):

(4) $$\alpha_{p^{\prime }}=\left|\left(\arctan\left(D\left(p,p^{\prime }\right)\right)-\beta_{i}\right)\right|.$$

**Figure 2:**
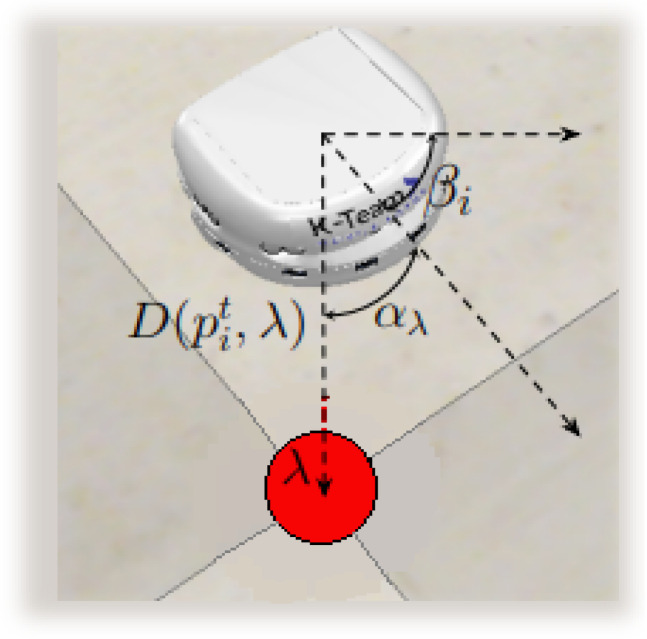
Illustration of angle $\alpha_{\lambda }$ between robot’s bearing $\beta_{i}$ and the orientation that the robot should adopt in order to reach landmark λ.

The angle $\alpha_{\lambda }$ is calculated as the normalized difference between the robot’s current orientation $\beta_{i}$ and the arctangent of the distance between the robot’s center of mass and the reference point. The normalization ensures that $\alpha_{\lambda }$ takes values in the range [−π,π]. This angle is illustrated in Figure 2.

**Figure 3:**
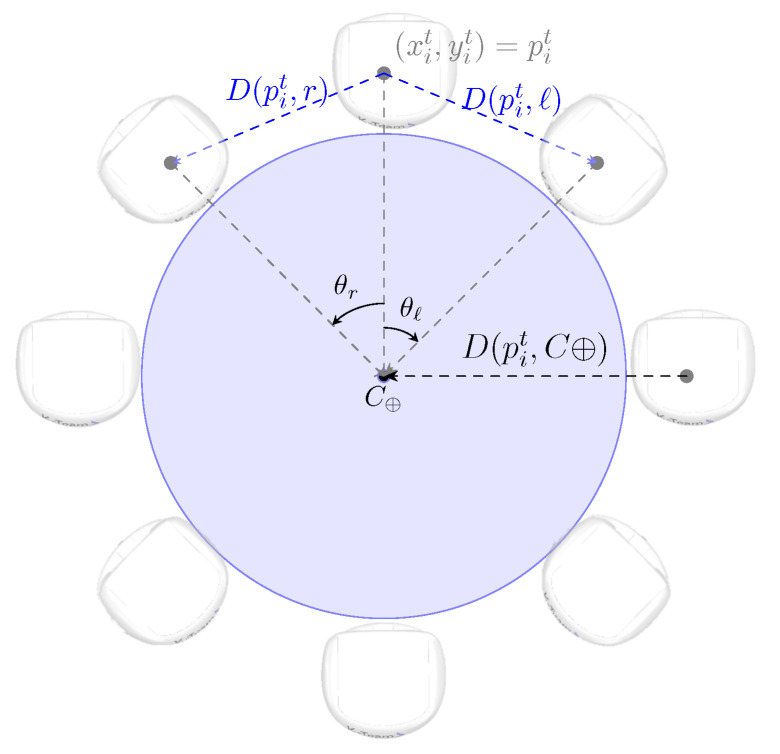
Illustration of the distances between robot *i* and object’s center of mass, $D(p_{i},C_{⊕})$; the distances between robot *i* and its right and left neighbors, $D(p_{i},r)$ and $D(p_{i},\ell )$; and angles between the bearing of robot *i* and those of the left and right neighbors, $\theta_{r}$, $\theta_{\ell }$, during the caging.

For the formation of the object caging, five other PID controllers are required. These are responsible for the following process variables: $D\left(p,p^{\prime }\right)=D\left(p_{i},r\right)$, $D\left(p,p^{\prime }\right)=D\left(p_{i},\ell \right)$, $D\left(p,p^{\prime }\right)=D\left(p_{i},C_{⊕}\right)$, $\theta_{r}$, and $\theta_{\ell }$. The distances $D(p_{i},C_{⊕})$, $D(p_{i},r)$, and $D(p_{i},\ell )$ are also expressed by Equation (3). $D(p_{i},C_{⊕})$ represents the distance between $p_{i}$ and the current position of the object’s center of mass $C_{⊕}$. The variables $D(p_{i},r)$ and $D(p_{i},\ell )$ represent the distance between $p_{i}$ and the current position of the center of mass of its neighbors on the right and left, respectively. The variables $\theta_{r}$ and $\theta_{\ell }$ represent the angles between the bearing of robot *i* and the current orientations of its neighbors on the right and left, respectively. It is worth noting that the ideal value for $\theta_{r}$ and $\theta_{\ell }$ is ϕ. Figure 3 illustrates the arrangement of these angles and distances in the caging formation.

Hence, the use of PID control for both the movement of the robots and the maintenance of the caging allows for the evaluation of all variables related to both movement and caging at each simulation step. Regardless of the position around the object that the robot occupies at a given step, the speed assigned to its motors, and consequently the force applied to the object, will be consistent with the function it needs to perform.

### 4.3. Robot Navigation Control

First of all, it is worth noting that we use the default Khepera-III model available in the used simulation platform [23]. It follows the same kinematic principles as a real robot, and fully complies with a real robot’s kinematics and dynamics as the model is properly calibrated according to physical parameters, such as wheel radius, wheelbase, and mass. The movement (linear and rotational) generated from wheel velocities matches the standard differential drive of the robot. This means that the fundamental motion behavior is aligned with a real robot’s kinematics.

The movement of the robots is developed using three state machines and ten PID controllers. The first state machine ${\mathrm{FSM}}_{1}$ controls the movement of the robots while the second ${\mathrm{FSM}}_{2}$ controls the orientation of the robots during the search and recruitment stages, and the third machine ${\mathrm{FSM}}_{3}$ controls the movement of the robots during the transport stage. It is important to note that the analysis of the finite state machines developed in this work does not reveal any deadlocks or infinite loops.

**Figure 4:**
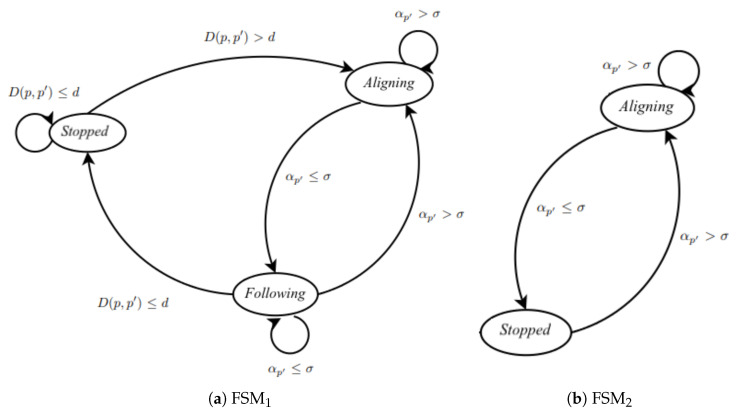
State transition diagram of ${\mathrm{FSM}}_{1}$ with PID for $D\left(p,p^{\prime }\right)=D\left(p,\lambda \right)$ and $\alpha_{p^{\prime }}=\alpha_{\lambda }$; ${\mathrm{FSM}}_{2}$ with PID for $\alpha_{p^{\prime }}=\alpha_{\lambda }$; and ${\mathrm{FSM}}_{3}$ with PIDs for $D\left(p,p^{\prime }\right)=D\left(p_{i},\lambda \right)$/$D(p_{i},r)$/$D(p_{i},\ell )$/$D(p_{i},C_{⊕})$, $\alpha_{p^{\prime }}=\alpha_{\lambda }$/$\theta_{r}$/$\theta_{\ell }$.

The state transition diagram of ${\mathrm{FSM}}_{1}$ is shown in Figure 4a. It is structured so that at each step, the variables related to the robots’ movement ($D\left(p,p^{\prime }\right)=D\left(p,\lambda \right)$ and $\alpha_{p^{\prime }}=\alpha_{\lambda }$) are analyzed using two PID controllers, thus verifying the distance and orientation of the robot in relation to its target reference point. The same applies to state machines ${\mathrm{FSM}}_{2}$ and ${\mathrm{FSM}}_{3}$, which have the same dynamics and whose state transition diagram is presented in Figure 4b. However, for ${\mathrm{FSM}}_{2}$, there is only one PID controller that controls the variable $\alpha_{p^{\prime }}=\alpha_{\lambda }$, while for ${\mathrm{FSM}}_{3}$, seven PID controllers are required to analyze the variables $D\left(p,p^{\prime }\right)=D\left(p_{i},\lambda \right)$, $D\left(p,p^{\prime }\right)=D\left(p_{i},r\right)$, $D\left(p,p^{\prime }\right)=D\left(p_{i},\ell \right)$, $D\left(p,p^{\prime }\right)=D\left(p_{i},C_{⊕}\right)$, $\alpha_{p^{\prime }}=\alpha_{\lambda }$, $\theta_{r}$, and $\theta_{\ell }$. So, these variables are inputs to PID controllers, which check their errors along the path and determine the necessary corrections. The state machine, upon receiving this information from the controllers, determines which variables need to be corrected by increasing or reducing the speed of the motors of each robot.

Algorithm 2 describes the state machine that controls the movement of the robots during the search and recruitment stages, as shown in Figure 4a.
**Algorithm 2** Move($D_{p^{\prime }},\alpha_{p^{\prime }}$) in robot *i***Require:** $p_{i},\beta_{i}$; **Ensure:** $Motor_{r},Motor_{\ell }$;
    **Compute**  $D_{p^{\prime }}=D\left(p,p^{\prime }\right)$ with Equation (3);
    **Compute**  $\alpha_{p^{\prime }}$ with Equation (4);
    **Normalize**  $\alpha_{p^{\prime }}$ so that $\alpha_{p^{\prime }}\in \left[-\pi ,\pi \right]$;
    PID($k_{p},k_{i},k_{d},T_{S},u_{sat},e_{b},r,D_{p^{\prime }}$); PID($k_{p},k_{i},k_{d},T_{S},u_{sat},e_{b},r,\alpha_{p^{\prime }}$);
    **Compute**  ${\mathrm{BraitSpeed}}_{r/\ell }$ with Braitenberg(sensors);
    **if** 
$D_{p^{\prime }}$
≤d
 **then**
        state :=stopped;
    **else**
        **if** state = stopped ∧ $D_{p,p^{\prime }}>d$ **then**
           state :=moving;
        **else**
           **if** state = moving ∧ $D_{p,p^{\prime }}>d$ **then**
               state :=moving
           **end if**
        **end if**
    **end if**
    **if** state = stopped **then**
        $Motor_{r}:=0.0$; $Motor_{\ell }:=0.0$;
    **else**
        **if** state = moving **then**
           $Motor_{r}:=\left(u_{D_{p^{\prime }}}+u_{\alpha_{p^{\prime }}}\right)+BraitSpeed_{r}$;
           $Motor_{\ell }:=\left(u_{D_{p^{\prime }}}-u_{\alpha_{p^{\prime }}}\right)+BraitSpeed_{\ell }$;
        **end if**
    **end if**


In this algorithm, the states *aligning* and *following* are combined into a single state called *moving*. In this configuration, the only parameter analyzed is the current distance between the robot and the reference point. When robot *i* is in the *stopped* state, it checks if $D(p_{i},\lambda )>d$. If true, robot *i* transitions to the *moving* state and remains there until $D(p_{i},\lambda )\leq d$, meaning the robot has reached the desired reference point.

Algorithm 3 presents the control of the robots’ orientation relative to the desired target position, as described in Figure 4b.
**Algorithm 3** Turn($\alpha_{p^{\prime }}$) at robot *i***Require:** $p_{i},\beta_{i}$; **Ensure:** $Motor_{r},Motor_{\ell }$;
    **Compute** $\alpha_{p^{\prime }}$ using Equation (4);
    **Normalize** $\alpha_{p^{\prime }}$ such that $\alpha_{p^{\prime }}\in \left[-\pi ,\pi \right]$;
    PID($k_{p},k_{i},k_{d},T_{S},u_{sat},e_{b},r,\alpha_{p^{\prime }}$);
    **if** 
$\alpha_{p^{\prime }}$
≤σ **then**
        state :=stopped;
    **else**
       **if** state = stopped ∧ $\alpha_{p^{\prime }}>\sigma$ **then**
          state :=moving;
       **else**
         **if** state = moving ∧ $\alpha_{p^{\prime }}>\sigma$ **then**
            state :=moving
         **end if**
       **end if**
    **end if**
    **if** state = stopped **then**
       $Motor_{r}:=0.0$; $Motor_{\ell }:=0.0$;
    **else**
       **if** state = moving **then**
         $Motor_{r}:=\left(+u_{\alpha_{p^{\prime }}}\right)$; $Motor_{\ell }:=\left(-u_{\alpha_{p^{\prime }}}\right)$;
       **end if**
    **end if**


This algorithm is used in the transportation stage and is quite similar to Algorithm 2. However, it uses only one PID controller, which controls the variable $\alpha_{p^{\prime }}$. Therefore, the correction applied to the motors by this algorithm controls only the orientation of the robots, unlike Algorithm 2, which controls both the orientation and the distance of the robot relative to the target reference point.

The state machine for the transportation stage is described through Algorithms 4–6, together with the state transition diagrams of Figure 4. Algorithm 4 presents the main algorithm of this state machine, showing the correct order of application. Algorithm 5 outlines the initialization steps, while Algorithm 6 displays the PID controllers applied during movement in the transportation stage. The purpose of Algorithm 5 is to invoke the PID controllers and obtain the control variables $u_{D_{\lambda }},u_{D_{r}},u_{D_{\ell }},u_{D_{⊕}},u_{\alpha_{\lambda }},u_{\theta_{r}}$, and $u_{\theta_{\ell }}$.
**Algorithm 4** Caging($D_{\lambda }$, $D_{r}$, $D_{\ell }$, $D_{⊕}$, $\alpha_{\lambda }$, $\theta_{r}$, $\theta_{\ell }$) at robot *i***Initialize** caging at robot *i*;**Apply** ${\mathrm{FSM}}_{1}$(d,ϵ);**Apply** ${\mathrm{FSM}}_{2}$($u_{D_{\lambda }},u_{D_{r}},u_{D_{\ell }},u_{D_{⊕}},u_{\alpha_{\lambda }},u_{\theta_{r}},u_{\theta_{\ell }}$);


**Algorithm 5** Initialize caging at robot *i***Ensure:** $u_{D_{\lambda }},u_{D_{r}},u_{D_{\ell }},u_{D_{⊕}},u_{\alpha_{\lambda }},u_{\theta_{r}},u_{\theta_{\ell }}$;
    **Compute** $D_{\lambda }=D\left(p_{i},\lambda \right);D_{r}=D\left(p_{i},r\right)$ with Equation (3);
    **Compute** $D_{\ell }=D\left(p_{i},\ell \right)$; $D_{⊕}=D\left(p_{i},C_{⊕}\right)$ with Equation (3);
    **Compute** $\alpha_{\lambda }$ with Equation (4); **Compute** $\theta_{r}$ and $\theta_{\ell }$;
    **Normalize** $\alpha_{\lambda },\theta_{r}$ e $\theta_{\ell }$ such that $\alpha_{\lambda },\theta_{r}$ e $\theta_{\ell }\in \left[-\pi ,\pi \right]$;
    **ControlTrsp** ($D_{\lambda },D_{r},D_{\ell },D_{⊕},\alpha_{\lambda },\theta_{r},\theta_{\ell }$);




**Algorithm 6** ControlTrsp($D_{\lambda },D_{r},D_{\ell },D_{⊕},\alpha_{\lambda },\theta_{r},\theta_{\ell }$) at robot *i***Ensure:** $u_{D_{\lambda }},u_{D_{r}},u_{D_{\ell }},u_{D_{⊕}},u_{\alpha_{\lambda }},u_{\theta_{r}},u_{\theta_{\ell }}$;
    PID($k_{p},k_{i},k_{d},T_{S},u_{sat},e_{b},r,D_{\lambda }$); PID($k_{p},k_{i},k_{d},T_{S},u_{sat},e_{b},r,D_{r}$);
    PID($k_{p},k_{i},k_{d},T_{S},u_{sat},e_{b},r,D_{\ell }$); PID($k_{p},k_{i},k_{d},T_{S},u_{sat},e_{b},r,D_{⊕}$);
    PID($k_{p},k_{i},k_{d},T_{S},u_{sat},e_{b},r,\alpha_{\lambda }$); PID($k_{p},k_{i},k_{d},T_{S},u_{sat},e_{b},r,\theta_{r}$);
    PID($k_{p},k_{i},k_{d},T_{S},u_{sat},e_{b},r,\theta_{\ell }$);



## 5. Algorithms for the Caging Strategy

The proposed collective transportation by caging is structured into four stages, as shown in Algorithm 7: Arena initialization, object search, robot recruitment, and object transport.
**Algorithm 7** Collective transport by cagingArena Initialization;**parallel** i:=0→ρ−1 **do** Object Search;**parallel** i:=0→ρ−1 **do** Robot Recruitment($r^{*},C_{⊕}$);**parallel** i:=0→ρ−1 **do** Object Transport;

The following section details the collective transportation by caging proposed in this work. In Section 5.1, the arena initialization algorithm is described. Section 5.2 details the algorithm of the search stage. Section 5.3 introduces the algorithms that sketch the recruitment stage. Section 5.4 describes the algorithm proposed for the transportation stage.

### 5.1. Arena Initialization

The steps of the arena initialization stage, described in Algorithm 8, depend on the total number of reference points Λ within the arena, the neighborhood function $\eta (\lambda_{i})$ between the reference points, and the total number of robots ρ. Based on these data, this algorithm determines the fundamental aspects for the correct operation of the proposed transport by object caging.
**Algorithm 8** Arena initialization**Require:** Λ, η(λ), ρ **Ensure:** $C_{⊕}$, $p_{i}^{0}$, $\mu_{i}$ for 0≤i<0ρ;
    **for** i:=0→Λ−1 **do Generate Randomly** $\lambda_{i}$ within arena;
    **for** i:=0→Λ−1 **do Define** $\eta (\lambda_{i})$;
    **Generate randomly** $C_{⊕}=\left(x_{⊕},y_{⊕}\right)$ within arena;
    **for** 
i:=0→ρ−1
** do**
       **Generate randomly** $p_{i}^{0}$ by the arena border;
       **for** j:=0→ρ−2 **do Define** $\mu_{ij}$;
    **end for**


The arena initialization stage is characterized by defining reference points throughout the arena, determining the neighborhood of each reference point by calculating the function $\eta (\lambda_{i})$. Additionally, this stage randomly determines the initial position of the center of mass of the object $C_{⊕}=\left(x_{⊕},y_{⊕}\right)$, and establishes the initial starting positions of the robots in the arena $p_{i}^{0}$ for 0≤i<ρ. Moreover, the arena initialization stage defines the order in which the robots are called during the recruitment stage through the matrix μ. This order is imposed so that there is no collisions between robots as this would cause the area to become cluttered.

Defining the reference points in the arena, which primarily allows for the discretization of the search space, and their neighborhoods is extremely important for the search stage. Their locations allow the robots to navigate the arena. The determination of the initial positions of the robots and the object is also of significant importance, as these definitions directly impact the search for the object. Another important definition made in this algorithm is matrix μ, as it is responsible for the recruitment order and consequently the positioning of the robots in the caging formation.

### 5.2. Object Search

The search stage aims to locate the object to be transported. In this stage, the robots are initialized in their initial positions, previously defined by Algorithm 8. The region where these robots are initially positioned is called the nest. After initialization, each robot selects a reference point to move towards. This selection can be random. However, in this work, only reference points belonging to the neighborhood of the robot’s current position, defined by the neighborhood function between reference points $\eta (\lambda_{i})$, can be selected. Upon reaching the selected reference point, the robot selects a new reference point, and this process iterates until the object is detected.

**Figure 5:**
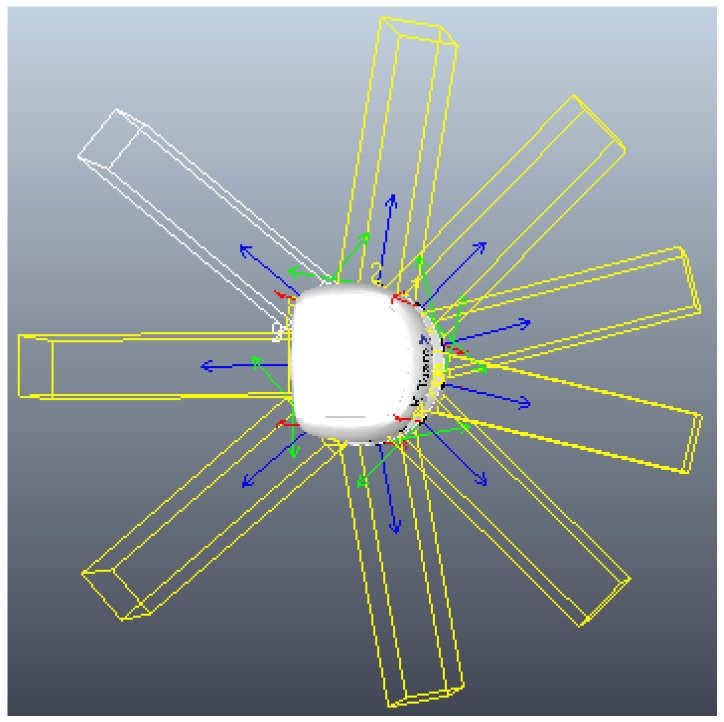
Sensor positions in Khepera-III.

So, the primary purpose of the search process is to identify the Cartesian position of the object in the arena. Therefore, the arrangement of proximity sensors along the Khepera-III robot [24] is crucial for the robot to quickly recognize the object as it approaches from any of its ends, as shown in Figure 5. Another important factor in this stage is the discretization of the arena, as it allows for better dispersion of the robots throughout the scenario and consequently greater efficiency in scanning the environment.

Algorithm 9 outlines the procedure for a robot *i* to search for the object. Initially, the first reference point λ is randomly selected as the destination for robot *i*. From this starting configuration, the robot moves from its current position $p_{i}$ towards the chosen reference point. The movement during the search is governed by a state machine and two PID controllers, which have the following process variables: $D\left(p,p^{\prime }\right)=D\left(p_{i},\lambda \right)$ and $\alpha_{p^{\prime }}=\alpha_{\lambda }$. This state machine and the PID controllers are encapsulated within the robot movement, described in Figure 4. At each simulation step, all sensors of robot *i* are evaluated. If any sensor detects the object, the robot halts its movement, is promoted to recruiter $r^{*}$, and initiates the recruitment phase. This phase involves sending messages from the recruiter robot to the swarm. Upon detecting the object, the recruiter sends type-0 messages containing the object’s center of mass position and the recruiter’s trajectory $T_{r^{*}}$ as the message content. Conversely, if the robot reaches the reference point λ without detecting the object, this point is added to the robot’s trajectory $T_{i}$, and a new reference point within the current vicinity is selected. This process repeats until robot *i* either detects the object or receives a message indicating that a recruiter has been identified. The procedure is iterated until the object is found.
**Algorithm 9** Object search at robot *i***Require:** *L*, η **Ensure:** $r^{*}$, $C_{⊕}$, $T_{r^{*}}$
    found:=; $r^{*}:=\infty$; msg:=∞; $n_{i}:=0$;
    **Select randomly** λ∈L;
    **repeat**
       **Compute** $D_{\lambda }=D\left(p_{i},\lambda \right)$ with Equation (3);
       **Compute** $\alpha_{\lambda }$ with Equation (4); **Move**($D_{\lambda },\alpha_{\lambda }$);
       **for** k:=0→s−1 **do**
         **if** $sensors\left[k\right].detect\wedge sensors\left[k\right].id=id_{⊕}$ **then**
            found:=; $r^{*}:=i$;
            **for** j:=0→ρ−1 **do**
               **if** $j\neq r^{*}$ **then Send** $msg\langle 0,r^{*},j,$$T_{r^{*}}$, $C_{⊕}\rangle$;
            **end for**
         **end if**
       **end for**
       **if** ¬found **then**
         **Receive** msg;
         **if** msg≠∞ **then**
            $r^{*}:=msg.origin$; $T^{*}:=msg.payload.T_{r^{*}}$;
            $C_{⊕}:=msg.payload.C_{⊕}$;
         **else**
            **if** $p_{i}\approx \lambda$ **then**
              **Append** λ to $T_{i}$; $n_{i}:=n_{i}+1$;
              **Select randomly** λ∈η(λ); $p_{i}:=\lambda$;
            **end if**
         **end if**
       **end if**
    **until** 
$r^{*}\neq \infty$


### 5.3. Robot Recruitment and Caging

Algorithm 10 outlines the main steps used for the recruitment stage. In this algorithm, it is determined whether robot *i* acts as the recruiter, meaning the first robot to identify the object. If robot *i* is designated as the recruiter, it follows the sequence of instructions whereby it recruits robots to help in the transport of the found object, as sketched in Algorithm 11. On the other hand, if robot *i* is not the recruiter, it initiates the sequence of instructions whereby it is recruited, as sketched in Algorithm 12.
**Algorithm 10** Robot recruitment ($r^{*},C_{⊕}$) at robot *i***if** $i=r^{*}$ **then**   Recruiter($C_{⊕}$);**else**   Recruited($C_{⊕}$);**end if**

**Figure 6:**
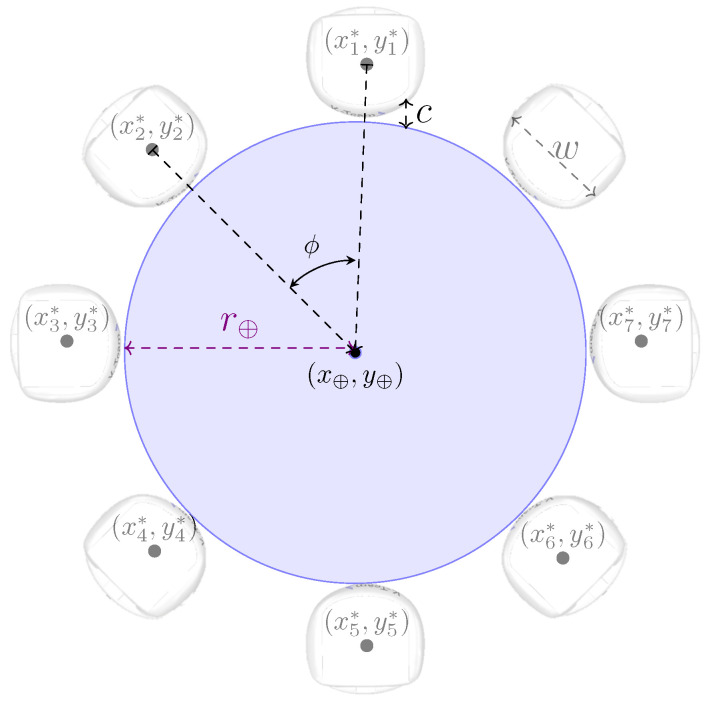
Illustration of initial caging.

Recruitment begins as soon as the search is completed, i.e., when one of the robots finds the object to be transported. Upon finding the object, the recruiter $r^{*}$ not only sends messages to the entire swarm indicating the current position of the object’s center of mass $C_{⊕}$ and its own constructed trajectory $T_{r^{*}}$, but also computes its position around the object, using Equation (5):

(5) $$\begin{array}{cc} x_{i}^{*}= & x_{⊕}+\left(\left(r_{⊕}+w\right)+c\right)\times \cos\left(i\times \phi \right);\\ y_{i}^{*}= & y_{⊕}+\left(\left(r_{⊕}+w\right)+c\right)\times \sin\left(i\times \phi \right),\; \end{array}$$

where *c* is a constant added to ensure that the robot does not touch the object when initially positioned, and *w* denotes the width of the robot. Figure 6 illustrates the initial positioning of the robots around the object.

Algorithm 11 describes the actions carried out by the recruiter robot. It navigates towards $p_{i}^{*}$ based on the state machine of Figure 4, with $D\left(p,p^{\prime }\right)=D\left(p_{i},p_{i}^{*}\right)$ representing the distance between $p_{i}$ and the initial caging position $p_{i}^{*}$ of robot *i*, and $\alpha_{p^{\prime }}=\alpha_{⊕}$. The distance is calculated using Equation (3) while the angle is computed using Equation (4). While the recruiter circles the object to find its initial position in the caging formation, the swarm approaches the position of the center of mass $C_{⊕}$ of the object, moving in that direction until reaching the approach distance Δ, estimated in Equation (6):

(6) $$\Delta =r_{⊕}+w/2+0.6.$$

After completing its positioning, robot $r^{*}$ sends a type-2 message to the swarm to indicate that it has finished and then summons the first robot as prescribed in $\mu_{r^{*}}$, waiting for that robot to reach its initial position in the caging formation. This process is repeated with the subsequent robots, according to the pre-established recruitment order in $\mu_{r^{*}}$, until the entire swarm is positioned, thus completing the caging of the object. Note that the recruitment is concluded when the process of object caging is completed.
**Algorithm 11** Recruiter($C_{⊕}$) at robot *i***Compute** $p_{i}^{*}$ with Equation (5); **Compute** $\alpha_{⊕}$ with Equation (4);**while** $\neg (p_{i}^{t}\approx p_{i}^{*})$ **do**   **Compute** $D_{*}=D\left(p_{i},p_{i}^{*}\right)$ with Equation (3); **Move**($D_{*},\alpha_{⊕}$);   **Compute** $p_{i}^{*}$ with Equation (5); **Compute** $\alpha_{⊕}$ with Equation (4);**end while****for** j:=0→ρ−2 **do**   $r:=\mu_{ij}$; **Send** (msg〈1,i,r,∅〉);   **repeat**     **Receive** msg;   **until** (msg.type=2)∧(msg.origin=r);**end for**

Algorithm 12 describes the actions of the recruited robots. Initially, robot *i* approaches the position of the object’s center of mass $C_{⊕}$ until the distance between the robot’s current position $p_{i}$ and the object’s center of mass $C_{⊕}$, i.e., distance $D(p_{i},C_{⊕})$, is at most the approach distance Δ, defined in Equation (6), or until it receives a type-1 message, indicating that it should proceed to its initial position in the caging formation. If robot *i* reaches the distance Δ before receiving this message, it waits. Upon receiving the type-1 message, robot *i* calculates its initial position $p_{i}^{*}$ around the object using Equation (5). Once $p_{i}^{*}$ is determined, the robot moves until its center of mass reaches approximately this position, and then sends a type-2 message to the recruiter, indicating that its initial positioning is complete.
**Algorithm 12** Recruited($C_{⊕}$) at robot *i***Compute** $D_{⊕}=D\left(p_{i},C_{⊕}\right)$ with Equation (3); **Compute** $\alpha_{⊕}$ with Equation (4);**while** $D_{⊕}>\Delta \vee \neg msg$ **do**   **Move**($D_{⊕},\alpha_{⊕}$); **Compute** $D_{⊕}=D\left(p_{i},C_{⊕}\right)$ with Equation (3);   **Compute** $\alpha_{⊕}$ with Equation (4); **Receive** msg;**end while****if** $D_{⊕}\leq \Delta$ **then Receive** msg;**if**$\left(msg.type=1\right)\wedge \left(msg.origin=r^{*}\right)$ **then**   **Compute** $p_{i}^{*}$ with Equation (5); **Compute** $\alpha_{⊕}$ with Equation (4);   **while** $\neg (p_{i}\approx p_{i}^{*})$ **do**     **Compute** $D_{*}=D\left(p_{i},p_{i}^{*}\right)$ with Equation (3);     **Move**($D_{*},\alpha_{⊕}$); **Compute** $p_{i}^{*}$ with Equation (5); **Compute** $\alpha_{⊕}$ with Equation (4);   **end while**   **for** j:=0→ρ−1 **do**     **if** j≠i **then Send** $\left(msg\langle 2,i,r^{*},\emptyset \rangle \right)$;   **end for****end if**

The recruitment process is heavily based on the exchange of messages between the robots. Therefore, extensive communication through signal exchange is a crucial and fundamental part of this phase. Another key aspect for the proper execution of the recruitment phase is the robots’ ability to navigate around the object and other robots as they approach them. This ability ensures that there are no collisions between robots during movement, including during the search phase, and that during the allocation of robots, one by one, around the object, errors from potential collisions are minimized. This ensures the correct and efficient encirclement of the object. The implementation of this capability was based on the Braitenberg vehicle presented by the CoppeliaSim platform in some of its example simulations. The algorithm used is provided in the simulation platform [9,23].

### 5.4. Object Transport

Once the caging is completed, the transportation phase begins. This phase, like the previous one, is characterized by extensive communication between the robots. For the transportation phase to start, all robots must confirm that they are in their correct initial positions and communicate this information to the others. Only after all the robots are aware that both they and the rest of the swarm are ready to begin the transportation, i.e., they are aware that the caging has been correctly finalized, is the subsequent transportation stage initiated.

The first step in the transport phase is identifying the path that the swarm will follow to transport the object from the location where it is found to the nest. This path is defined by the recruiter, meaning it is the reverse of the path that the recruiter followed during the search phase, ensuring the swarm passes through each $\lambda_{i}$ traversed by the recruiter until reaching the nest. The trajectory, composed by the reference points traversed, is stored during the search phase, and the recruiter’s path is chosen because it is the first robot to reach the object, making it likely the shortest path between the object’s initial position and the nest.

After receiving the path, the robots identify the $\lambda_{i}$ to which the object should be transported, then individually orient themselves in the direction to be followed. After the swarm communicates and collectively confirms that all robots are correctly oriented, they begin applying forces to the object, moving it until it reaches that $\lambda_{i}$. Upon arrival, they check whether the current location is the final point of the path; if so, the transportation is completed. Otherwise, following the same processes of communication, orientation, and movement, they transport the object from $\lambda_{i}$ to the next waypoint in the constructed path.

The main challenge of the transportation phase is maintaining the caging along the route, as well as each robot correctly applying forces to the object according to its position at each simulation step. Along the path, the same robot may be positioned at the back of the object, applying force to move it, or at another moment in the same simulation, due to the orientation towards $\lambda_{i}$, the same robot might be on the side or even in front of the object. In this case, the force it applies would aim to maintain the encirclement, keep the object oriented, and provide resistance, acting as a counterforce to the movement, enabling coordinated movement of the object and minimizing potential deviations from a straight trajectory. To implement this without assigning specific functions to each robot, which would require repositioning the robots around the object at each simulation step, a state machine and PID controllers are used to maintain the caging, as described in Algorithm 4.

Algorithm 13 presents the transportation routine for robot *i*. Initially, robot *i* waits to receive type-2 messages from all ρ robots, as receiving these messages indicates that the encirclement is complete and transportation can begin. The trajectory $T_{r^{*}}$ to be used for transporting the object is the same as the one followed by the recruiter robot $r^{*}$, but in reverse order. In this case, the robot’s orientation is achieved using the state machine presented in Algorithm 3 and a PID controller, with $\alpha_{p^{\prime }}=\alpha_{\lambda }$. After orienting itself in the direction of λ, robot *i* informs the swarm by sending a type-3 message about the completion of its orientation process. It then waits for confirmation from the swarm through the same type of message, indicating that the entire swarm has completed this process. Once all robots are oriented in the direction of the reference point, the simultaneous movement of the swarm begins, resulting in the transportation of the object.

The movement of robot *i* during transportation follows Algorithm 4, and this movement is considered complete when the robot’s current position $p_{i}$ matches the position it should reach when the object arrives at the reference point $p_{i}^{+}$. The position $p_{i}^{+}$ is calculated similarly to $p_{i}^{*}$, as presented in Equation (5). When $p_{i}$ reaches $p_{i}^{+}$, robot *i* sends type-4 messages to the entire swarm, informing them that the reference point has been reached. It then waits for confirmation that the entire swarm has also reached λ through the same type of message. This process is iterated until the entire trajectory $T_{r^{*}}$ is completed, and consequently, the transportation of the object is concluded.
**Algorithm 13** Object transport at robot *i*j:=0;**while** j<ρ **do**   **Receive** msg;   **if** (msg.type=2)∧(msg.origin=j)∨j≠i **then** j:=j+1;**end while****for** j:=n−1→0 **do**   $\lambda :=T_{r^{*}j}$;   **repeat**     **Compute** $\alpha_{\lambda }$ with Equation (4); Turn($\alpha_{\lambda }$);   **until** $\beta_{i}\approx \alpha_{\lambda }$   **for** j:=0→ρ−1 **do**     **if** j≠i **then Send** msg〈3,i,j,∅〉;   **end for**   **while** k<ρ−1 **do**     **Receive** msg;     **if** (msg.type=3)∧(msg.origin=k)∨k≠i **then** k:=k+1;   **end while**   **repeat**     **Compute** $D_{\lambda }$=$D(p_{i},\lambda )$, $D_{r}$=$D(p_{i},r)$ with Equation (3);     **Compute** $D_{\ell }$=$D(p_{i},\ell )$,$D_{⊕}$=$D(p_{i},C_{⊕})$ with Equation (3)     **Compute** $\alpha_{\lambda },\theta_{r}$, $\theta_{\ell }$ with Equation (4); **Caging**($D_{\lambda }$, $D_{r}$, $D_{\ell }$, $D_{⊕}$, $\alpha_{\lambda }$, $\theta_{r}$, $\theta_{\ell }$);   **until** $p_{i}\approx p_{i}^{+}$   **for** j=:0→ρ−1 **do Send** msg〈4,i,j,∅〉;   k:=0;   **while** k<ρ−1 **do**     **Receive** msg;     **if** (msg.type=4)∧(msg.origin=k)∨(k≠i)**then** k:=k+1;   **end while****end for**

## 6. Implementation Issues and Setup

In this work, the CoppeliaSim platform [23] is adopted as the simulation environment for the development of the proposed transport strategy. This choice is motivated by the fact that the platform includes models of physical robots in its library, allowing for the future implementation of the developed simulations in physical models. However, for the physical implementation of this work to be feasible, certain factors must be considered. Due to the need for continuous communication between the robots, the physical implementation of the proposal would require the arena to be defined in such a way that signals between the robots could be transmitted and received reliably. Additionally, in a real-world scenario, communication and computation times for the robots show significant disparity compared to the values obtained in simulated environments. Therefore, a meticulous analysis of these parameters is crucial for the effective transition of this implementation to a physical context, as it is extremely important in the proposed strategy that the robots perform certain processes simultaneously.

Lua is used in this implementation because it allows for the parallel execution of the robots’ code within the platform. Each robot has its own code and executes it independently of the others. The Khepera-III robot [24] without the grippers is used due to its rounded front, which allows for good contact between the front of the robot and the contour of the object, a critical detail for the encirclement and transportation phases.

The arena is the setting where simulations are carried out on the simulation platform. In this implementation, arenas with dimensions of 5 m × 5 m and 10 m × 10 m are used, and they are discretized with steps of 0.5 m and 1 m. The arenas are flat. The object to be transported is cylindrical, and its dimensions vary according to the case study presented in Section 7.

As detailed in Section 4.3, the movement of the robots is implemented using state machines and PID controllers. Table 1 presents the setting of the proportional, integral, and derivative components used in the PID controllers. The values applied to the proportional, integral, and derivative constants are adjusted through various simulations to achieve the desired control.

Table 2 displays the physical characteristics of the robots, such as the width *w* and mass *m*; and the values of the parameters used in the equations, such as the minimal inter-robot distance in the caging formation ε, the distance between the object and the robot when initially positioned *c*, and the approach distance Δ in the recruitment stage. Note that ε is set to half the robot’s width *w*. This setting was validated practically via an empirical local sensitivity analysis. Additionally, Table 2 includes the setting up of the tolerance constants used in the state machines, in terms of distance *d* and orientation σ. Recall that *M* and $r_{⊕}$ denote the object’s mass and radius, respectively, and are defined later as they vary from one case to another.

## 7. Performance Results

This section aims to present and analyze the performance of this work in the search, recruitment, and transport stages. All simulations were run on a computer with an Intel Core i9 and 128 GB. Section 7.1 describes the impacts of the search and recruitment stages, including the initial caging of the robots around the object. Section 7.2 provides and discusses a performance evaluation of the transport stage through three case studies. Section 7.3 offers a statistical analysis to demonstrate the confidence of the simulation results. In Section 7.4, we provide a comparison with an existing work.

### 7.1. Results Regarding Search and Recruitment

The search stage was implemented by defining a neighborhood among the reference points in the arena. From the current reference point, the robot randomly selects a new reference point within a predefined neighborhood to which it should move. The neighborhood function $\eta (\lambda_{i})$ is implemented based on Equation (7):

(7) $$\eta \left(\lambda_{i}\right)=\left\{\lambda_{i,j+1},\lambda_{i+1,j+1},\lambda_{i-1,j+1}\right\},$$

where *i* and *j* represent the row and column of the arena cell intersections at which the reference point is located. The reference points are arranged in a matrix form in the arena. In this way, the neighbors of each reference point belong to the next column of reference points, allowing the robot to continuously advance in its search through the arena. Meanwhile, the random selection among reference points within the predefined neighborhood ensures greater dispersion of the robots throughout the arena during the search stage.

**Figure 7:**
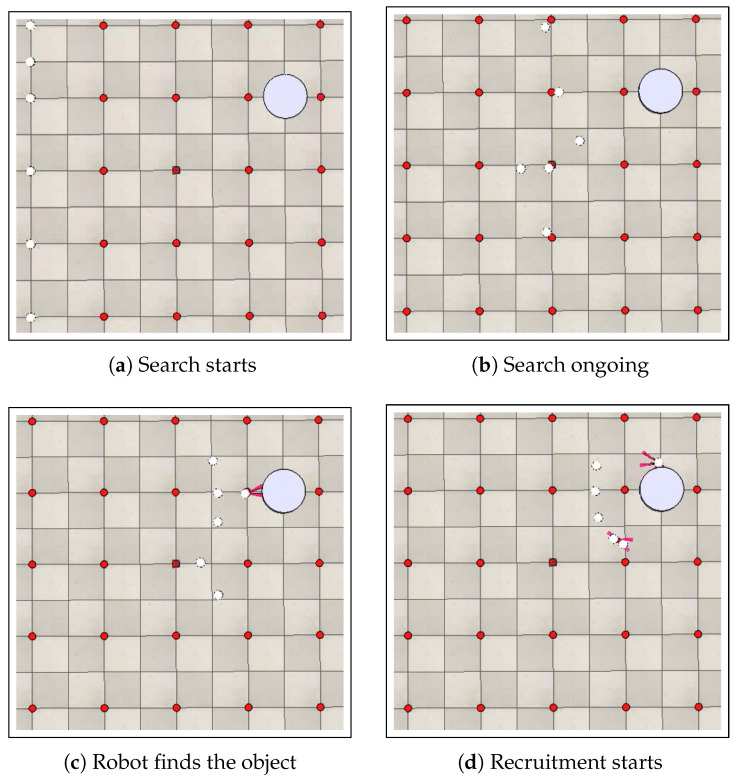
Simulation snapshots during the search and recruitment stages.

Figure 7 presents the search and recruitment stages for a swarm composed of six robots (ρ=6) in an arena with dimensions of 5 m × 5 m and a discretization step of 1 m.

The performance analysis of the search stage was conducted using two case studies, four experiments, and ten simulations for each experiment, totaling forty simulations. Table 3 presents the results obtained for the search stage using six robots. The results are obtained for two arenas: one of 5 m × 5 m and another of 10 m × 10 m. Each of these arenas is discretized with two different steps: 0.5 m and 1 m. The presented results are average times of the search stage ($A_{TS}$) together with the standard deviation, confidence interval and relative error, obtained for 10 simulations in each experiment, totaling forty simulations. We consider a 95% confidence level. It can be noted that the error is about 2% for all cases, which is lower than 5% and thus the results are considered precise.

The analysis of the performance in the search and recruitment stages is conducted by examining the average time required for these stages under different arena discretization steps. It is observed that in the search stage, the discretization step of the arena has a direct impact on this quantity. This occurs because the closer the robot is to the reference point, the lower its speed, allowing for better adjustment of its route. Therefore, the smaller the discretization step, the lower the speed reached by the robots during the search stage. On the other hand, the average time required for the execution of the recruitment stage is independent of the arena discretization. This stage is affected by the distance between the robots and the object when it is detected. The execution time of the recruitment stage is also impacted by the pre-established initial positioning order (μ).

**Figure 8:**
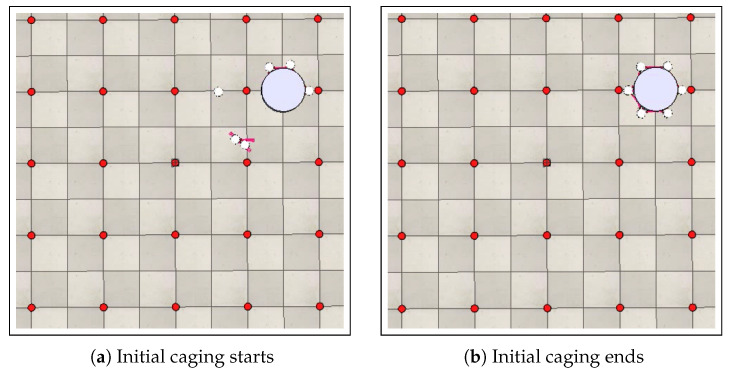
Simulation snapshots during initial object caging.

Once the first step of the recruitment, in which the robot approaches the found object, is completed, the second step, which deals with initial positioning of the swarm around the object, begins. In this step, a pre-established recruitment order $\mu_{r}^{*}$ is adopted. In other words, for each robot *i* that becomes the recruiter $r^{*}$, there is a pre-established sequence of robot calls for initial positioning. The recruiter robot initially calls its left and right neighbors. After the positioning of these neighbors, the robots that occupy the positions immediately adjacent to the recruiter’s neighbors are also called. This process is repeated until the entire swarm is correctly positioned, thereby completing the object’s caging. It is noteworthy to point out that a predefined initial positioning order, represented by μ, is used. This is aimed at preventing possible collisions between robots, as well as between the robots and the object, during the initial positioning stage. Figure 8 shows the initial positioning stage. Once the initial positioning is completed, the object’s caging is also completed, as shown in Figure 8b.

### 7.2. Results Regarding Transport

**Figure 9:**
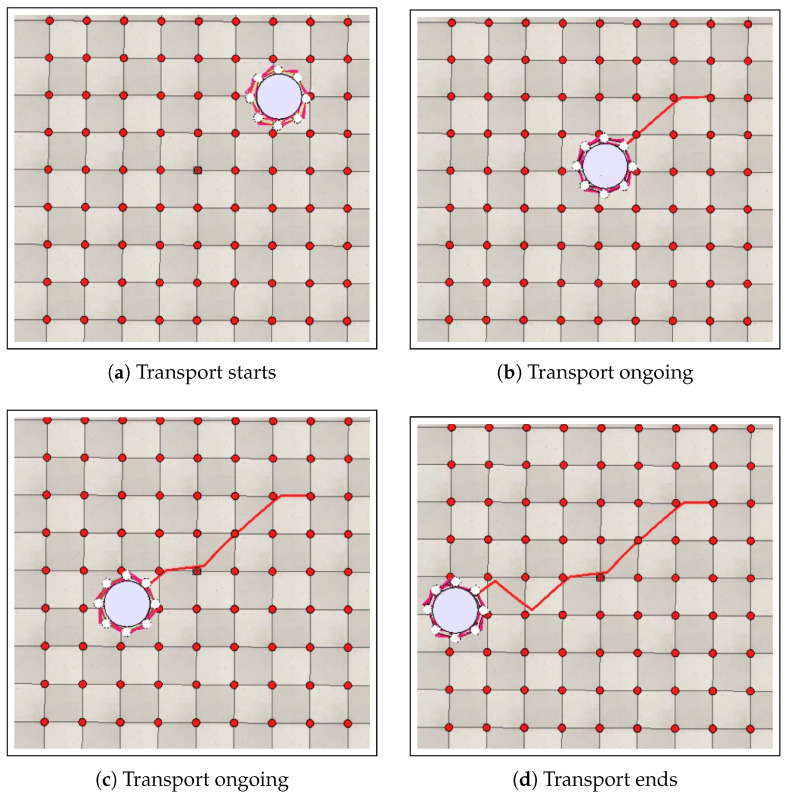
Simulation snapshots during the transport stage.

The performance analysis of the transport stage was based on three case studies, in which the impacts on the results were evaluated for different numbers of robots, objects with varying dimensions, and arenas with different discretization steps. Section 7.2.1 evaluates the results for swarms with different numbers of robots. Section 7.2.2 analyzes the results of transporting cylindrical objects with different dimensions, and Section 7.2.3 discusses the results obtained for arenas with different discretization steps. Figure 9 illustrates the transport stage for a swarm composed of eight robots (ρ=8) in an arena with dimensions of 5 m × 5 m and a discretization step of 0.5 m.

#### 7.2.1. Robot Number Impact

To analyze the impact of the number of robots on object transport, three swarms of different sizes were defined, each with a predetermined number of robots. The swarms were set with the following robot counts: ρ=6, ρ=7, and ρ=8. For this implementation, an arena with dimensions of 5 m × 5 m and a discretization step of 1 m was used. The object had a cylindrical shape with a radius of $r_{⊕}$ = 0.3 m and a mass of M = 0.3 kg. The complete simulations for this case study are available at: linktr.ee/numeroderobos, accessed on 1 August 2025.

The trajectory error during the object’s transport is analyzed for the three different robot numbers, as well as the time required to complete all the stages of collective transport by encapsulation, including search, recruitment, initial positioning, and transport. The error is calculated based on the absolute difference between the trajectory that the object should follow during transport, $T_{r^{*}}$, and the actual path traveled by the object’s center of mass. Specifically, the minimum distance to be covered based on the trajectory $T_{r^{*}}$ is calculated by summing the distances between the reference points belonging to $T_{r^{*}}$. Then, the distance traveled by the object’s center of mass during transport is calculated by summing the distances between the positions reached by the object’s center of mass along the way when the swarm considered it had reached the desired reference point. Thus, by calculating the absolute difference between these paths, it is possible to determine the error in the trajectory traveled by the object’s center of mass. The calculation of the ideal path is obtained from Equation (8):

(8) $$IP=D\left(C_{⊕},T_{r^{*}0}\right)+\sum_{j=0}^{n-1}D\left(T_{r^{*}j},T_{r^{*}j+1}\right),$$

where *n* represents the size of the trajectory vector $T_{r^{*}}$. Distance $D(C_{⊕},T_{r^{*}0})$ is the distance between the object’s center of mass at the position where it was found and the position of the first reference point to which the object should be transported. Distance $D(T_{r^{*}j},T_{r^{*}j+1})$ represents the distance between consecutive reference points along the trajectory $T_{r^{*}}$ to be followed during transport. The calculation of the actual path traveled by the object’s center of mass is based on Equation (9):

(9) $$AP=D\left(C_{⊕},C_{⊕}^{T_{r^{*}0}}\right)+\sum_{j=0}^{n-1}D\left(C_{⊕}^{T_{r^{*}j}},C_{⊕}^{T_{r^{*}\left(j+1\right)}}\right),$$

where distance $D(C_{⊕}^{T_{r^{*}j}},C_{⊕}^{T_{r^{*}\left(j+1\right)}})$ represents the distance between the subsequent positions reached by the object’s center of mass when, during the transport, the swarm assumes that the object’s center of mass has reached the desired reference point. The error calculation is obtained from the absolute difference between the ideal path and the actual path traveled by the object’s center of mass, as shown in Equation (10):

(10) E=|AP−IP|.

**Figure 10:**
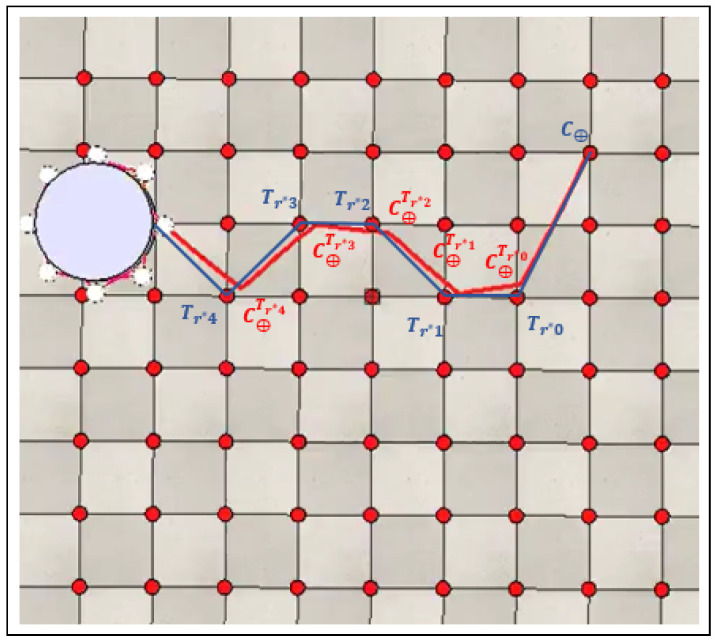
Illustration of the actual and ideal paths: AP vs. IP, wherein AP is depicted red the blue line and IP is represented by the blue line.

Figure 10 shows the ideal path IP (blue) and the actual path AP traveled by the object’s center (red), highlighting the error *E* between these paths.

A useful metric to evaluate efficiency in terms of both overall time and precision, which denotes the trajectory accuracy in a collective transport task is the efficiency score (ES), defined as

(11) $$ES=\frac{P}{{\left(T_{norm}\right)}^{\omega_{T}}\cdot {\left(E_{norm}\right)}^{\omega_{E}}},$$

where *P* represents the length of the transport path; $T_{norm}$ is the normalized overall execution time taken to complete the whole task; and $E_{norm}$ is the normalized trajectory error, which denotes the mean deviation from the ideal path, normalized to the object’s size or path length. The coefficients $\omega_{T}$ and $\omega_{E}$ weigh the importance of time vs. accuracy. In the presented results, we give the same importance to time and accuracy, setting up both factors with the same value: $\omega_{T}=\omega_{E}=0.5$. Moreover, we use the z-score normalization method and apply logarithmic scaling when necessary [25]. Note that a lower time and lower error result in a higher efficiency score, indicating better performance. If either time or error is high, the score drops, revealing inefficiency due to slowness or inaccuracy. The metric is unit-independent. Taking into account the length of the ideal path makes the metric suitable for comparing different strategies or swarm configurations.

In this case study, where the impact of the number of robots on the path error is analyzed, three experiments were conducted, with ten simulations per experiment, totaling thirty simulations. Each experiment included a specific number of robots: 6, 7, and 8. The arena had dimensions of 5 m × 5 m with a discretization step of 1.0 m. The transported object had a mass of 0.3 kg and a radius of 0.3 m.

We report the best cases in terms of time requirements. Table 4 presents the performance results for these simulations, wherein TT and OT stand for transport time and overall time, respectively. It can be noted that the percentage error for the three different numbers of robots is approximately 4%, while the error in meters is around 0.17 m. This indicates that the path error remains roughly constant in this implementation, regardless of the number of robots in the swarm. As for the execution time, it is observed that the more robots there are in the swarm, the longer it takes to complete the transportation by caging. This is due to the initial positioning of the robots around the object. The more robots there are, the more time is required for them to be correctly positioned around the object, thereby completing the caging process. During this stage, the robots must maneuver around the object and avoid collisions with both the object and the other robots, which significantly increases the time needed for the robots to position themselves correctly in their initial caging positions. It is safe to conclude that increasing the number of robots does not lead to a reduction in the execution time of the transportation stage, as these robots operate at their maximum constant speeds. Note that although, according to Equation (1), each robot should exhibit a continuously increasing acceleration, resulting in a constantly increasing velocity, the physical limitations of the robots impose a maximum velocity. Consequently, upon reaching this limit, the robots operate continuously at their maximum velocity. This is confirmed by the efficiency score, which is the highest for ρ=6, which is the minimal number of robots, required to transport the object, according to its characteristics.

#### 7.2.2. Impact of Object Characteristics

This case study aims to examine the impact of the physical dimensions of the object on transportation, specifically to determine whether these dimensions significantly affect the trajectory error of the object’s center of mass. Three experiments are conducted, with one simulation per experiment.

For this case study, the arena is kept with dimensions of 5 m × 5 m and a discretization step of 1 m. The swarm is set to eight robots, ρ=8, and the object has three different dimensions. The complete simulations for this case study are available at: linktr.ee/objetodimensoes, accessed on 1 August 2025. We report the best cases in terms of time requirements. Table 5 presents the performance results obtained for these simulations. The calculation of the ideal path IP is obtained using Equation (8), the calculation of the real path is obtained using Equation (9), and the error is calculated using Equation (10). Table 5 presents the obtained results for this case, wherein TT and OT stand for transport time and overall time, respectively.

Based on the results of Table 5, it is noted that the percentage error in the trajectory, in this case, is approximately 3.6%, while the error in meters is around 0.16 m. It is also safe to conclude that regardless of the object’s dimensions, the path error remains approximately constant, highlighting the robustness of the proposed methodology. It is also observed that increasing the object’s mass does not result in a longer time being required for the execution of the transportation stage, as these robots operate at their maximum constant speeds. In other words, the robots maintain a constant speed, regardless of the object’s mass. The ES analysis shows that efficiency is not linear with size or mass, as the medium case performed worse than both small and large objects, suggesting a non-monotonic behavior. The highest efficiency score is achieved for the configuration $r_{⊕}$ = 0.30 m and *M* = 0.30 kg, indicating the best balance between transport time and path precision in this case.

#### 7.2.3. Arena Discretization Impact

In order to analyze the impact of arena discretization on collective transportation by enclosure, simulations similar to those described in the previous sections were conducted, maintaining the same arena dimensions of 5 m × 5 m, but using two different discretization steps: 0.5 m and 1.0 m. The arena with a discretization step of 0.5 m consists of 81 reference points, while the arena with a discretization step of 1.0 m consists of 25 reference points. The complete simulations are available at: linktr.ee/discretizacaodaarena, accessed on 1 August 2025.

First, we investigate whether there is an impact on the path error obtained for arenas with different discretization steps while varying the number of robots. Six experiments are conducted, with ten simulations per experiment, totaling sixty simulations. We report the best cases in terms of time requirements. Table 6 presents the performance results for swarms composed of different numbers of robots. From the analysis of these results, it can be observed that the smaller the discretization step of the arena, the greater the path error. Similar to the study evaluating the impact of the number of robots for the arena with a discretization step of 1.0 m, a larger number of robots results in a longer execution time for collective transportation by enclosure, due to the impact of the initial positioning stage. So, the more robots there are, the more time is required for them to correctly position themselves around the object, thus caging it. In this case, the object has a radius of $r_{⊕}$ = 0.3 m and a mass of *M* = 0.3 kg.

Therefore, the percentage error for the arena with a discretization step of 0.5 m is approximately 6.2%, while the error in meters is approximately 0.28 m. This represents a significant increase compared to the arena with a discretization step of 1.0 m, where the percentage error is approximately 4%. This occurs due to the larger number of reference points between the initial position of the object in the arena and the target position to which it is transported. Therefore, the smaller the discretization step is, the more reference points are involved in the path to be traversed. Consequently, more local errors are produced, leading to higher accumulated error along the path. The efficiency score confirms the conclusion as the highest scores are obtained for the discretization step 1.0. The highest efficiency is achieved in the case ρ=7 and step=1.0, yielding the best balance of transport time and precision relative to the ideal path length.

Second, we investigate whether there is an impact on the path error obtained for arenas with different discretization steps while varying the characteristics of the object. Six experiments are conducted, with ten simulations per experiment, totaling sixty simulations. We report the best cases in terms of time requirements. Table 7 presents the performance results for swarms composed of eight robots and objects with different dimensions. It is observed that, for the arena with a discretization step of 0.5 m, the percentage error of the path is approximately 6.3%, while the error in meters is approximately 0.29 m. Comparing these errors with those obtained for the same cases with a discretization step of 1 m, an increase is observed.

So, it is safe to conclude that the smaller the discretization step of the arena, the greater the accumulated error during the path. Consequently, as the discretization step decreases, more reference points are involved in the path to be traversed, leading to more local errors and consequently a higher accumulated error during the journey. Furthermore, for arenas with the same configuration, the percentage error of the object’s center of mass trajectory is approximately constant regardless of the number of robots or the object’s dimensions. Hence, the trajectory error of the object’s center of mass is affected only by the arena’s discretization step. The highest efficiency scores are achieved for discretization with a step of 1.0.

### 7.3. Statistical Confidence Results

In order to show the statistical confidence in the results obtained during the simulations, we present the mean values, the standard deviation, and the confidence interval, as well as the relative error under a 95% confidence level, considering the ten conducted simulations. Here, we consider only the results obtained for the case with a transported object of $r_{⊕}$ = 0.3 m and *M* = 0.3 kg, but for three swarm sizes and two arena discretizations. Due to space constraints, we present only these representative cases, as the remaining ones, i.e., for $r_{⊕}$ = 0.4 m and *M* = 0.7 kg, and $r_{⊕}$ = 0.45 m and *M* = 1.0 kg, exhibit similar behavior. Table 8 and Table 9 show the statistical results for the transport path with overall times and path error with efficiency scores, respectively. It can easily be noted that the statistical relative errors (REs) for all cases are below 5% and thus the simulation results are considered precise.

### 7.4. Performance Comparison: Pushing vs. Caging

For further assessment, we compare the performance of the pushing strategy to the proposed caging strategy. For this, we consider the work presented in [26]. Therein, a collective transport of an object by a swarm of robots is proposed using the pushing strategy. Also, different search strategies are investigated. In addition to random search, the search stage is optimized exploring an ant colony-based technique called Ant Colony System [27]. The implementation is made using the Robotarium simulator platform [28]. The case studies utilized in [26] are slightly different from the one used in this work. They consist of some differences in the arena dimensions, the number and disposition of the waypoints in the arena, and in the definition of their neighborhood. We list all these data in Table 10. We selected the neighborhood the closest to the one used here, wherein the robots navigate by selecting the waypoints in forward-only fashion. Note that the simulations run on computers with similar characteristics.

Table 11 presents the lengths of the ideal and actual paths, IP and AP, respectively, based on which we compute and present the imposed error *E* and the efficiency scores *ES*s, and we also report on the transport and overall times, TT and OT, respectively, based on which we compute and present the efficiency scores *ES*s. In the case of results taken from [26], we show the values obtained for these metrics in the case of a random and optimized search, and in the case of the results of the proposed work, we show the results for two discretization steps.

Considering the results of Table 11, it is safe to conclude that the pushing strategy adheres more to the ideal path than the caging strategy, as the errors are smaller in the first case. The pushing strategy is actually about 10× more precise than the caging strategy. However, this comes at a price, as we can notice that the transport time when using the pushing strategy is much higher than its counterpart for the caging strategy. It is actually about 5× higher when the arena is discretized using a 0.5 step and about 8.5× higher when a 1.0 step is used. Moreover, even though the search stage in [26] is optimized, the overall time therein is about 2× and 3× higher than the overall time requirements in this work for the arena discretizations of 0.5 m and 1 m, respectively. Additionally, based on the achieved efficiency scores, we can confirm that the caging strategy with the discretization step of 1.0 is very efficient, due to quicker completion and moderate error, making it suitable when both time and accuracy are critical. Furthermore, the pushing strategy, in the case of the optimized search, is effective but may take more time or produce higher error relative to the path length.

## 8. Conclusions

In this work, the problem of cooperative transport by caging is addressed. An algorithm is designed to enable a swarm of robots to collectively transport a cylindrical object by caging it. The proposed method makes use of state machines and PID controllers to control and maintain the enclosure of the object throughout the entire transport trajectory. The obtained results demonstrate that the proposed method is capable of maintaining the enclosure of the object along the entire trajectory over which it must be transported. The algorithm includes several stages in addition to the transportation itself. These stages are the search for the object in the arena by the robot swarm, the recruitment of the swarm to the object’s detection area, the initial positioning of the robots around the object to enclose it, and the actual transportation.

The performance evaluation shows that the proposed caging strategy is effective in collectively transporting objects by a swarm of robots. Comparing the pushing strategy to the caging strategy, we show that the former is more precise regarding the traversed path during transport while the latter is much more efficient in terms of transport time, but less precise.

The proposed approach for the search stage does not ensure that the first robot to detect the object has taken the shortest path from its initial position to the object’s location. Since this path is traveled in reverse during the transport phase, it needs to be optimized. Therefore, the exploration of trajectory optimization algorithms is necessary to ensure an optimized path. In this work, the object is cylindrical. Nonetheless, the developed strategy and underlying algorithm can be applied to objects of various shapes, provided that a mathematical model is constructed, similar to the one proposed in this work, for the specific desired shape. In other words, it is necessary to establish the parameters required for caging the desired object, regarding angles and distances, in order to allow for the calculation of the initial positioning of the robots around the object to enclose it. By identifying these critical parameters, which constitute the process variables, that must be controlled during transport to ensure enclosure, along with the initial positioning equation of the robots’ positions, it becomes possible, with minor adaptations to the algorithm, to use it for collective transport by enclosure of non-cylindrical objects. The proposed strategy can also be applied in non-planar arenas and also in fluid or aerial environments. However, in these cases, physics laws regarding navigation and applied forces, such as friction or inertia, need to be revisited and the model of transport adjusted. Additionally, a promising direction for future research involves the search, recruitment, and transport of multiple objects by multiple robot swarms. This would explore the system’s ability to handle more complex and diverse tasks, which could have significant implications in real-world applications. Moreover, the implementation of this proposal using real robots is intended, which will introduce further challenges related to hardware limitations, and environmental factors such as communication bandwidth and data loss, as well as real-time decision making.

Looking ahead, we aim to transition the proposed caging-based swarm transport methodology to real-world industrial applications. To achieve this, several key challenges must be addressed. Future work will focus on accommodating robot heterogeneity by developing role-assignment and control strategies that leverage the diverse capabilities of individual robots. Additionally, improving the swarm’s performance in obstacle-rich environments will require the integration of real-time localization, mapping, and collision avoidance systems. Cost-efficiency will also be a critical factor, motivating the use of low-cost, modular robot platforms capable of maintaining effective caging behavior with minimal hardware complexity. Initial real-world validation will be conducted in structured settings such as warehouses, allowing the swarm’s coordination and transport capabilities to be gradually tested and refined under controlled conditions. In the case of real-world applications, it is also important to extend the methodology to incorporate fault-tolerant mechanisms, ensuring the swarm can maintain effective transport even in the presence of individual robot failures or communication disruptions.

## Figures and Tables

**Table 1:** Proportional, integral, and derivative constants used to set up the PID controllers.

PIDs	$\alpha_{p^{\prime }}$	$D(p_{i},\lambda )$	$D(p_{i},\ell )$, $D(p_{i},r)$	$\theta_{\ell }$, $\theta_{r}$	$D(p_{i},C_{⊕})$
$k_{p}$	1	1	0.3	0.3	1
$k_{i}$	0.01	0.01	0.01	0.01	0.01
$k_{d}$	0.01	0.01	0.1	0.1	0.1

**Table 2:** Robot characteristics, used constants, and thresholds.

*w* (m)	*m* (kg)	*a* (m/s^2^)	ε (m)	*c* (m)	Δ (m)	*d* (m)	σ (°)
0.122	0.65	0.00354	0.061	0.01	$r_{⊕}+0.661$	0.05	3

**Table 3:** Performance results of the search stage for different scenarios using 6 robots.

Arena (m)	Step (m)	$A_{\mathrm{TS}}$ (s)	$\sigma_{\mathrm{TS}}$	${\mathrm{CI}}_{\mathrm{TS}}$	RE (%)
5×5	0.5	70.56	2.334	[68.886, 72.226]	2.366
	1.0	60.12	1.841	[58.803, 61.437]	2.190
10×10	0.5	222.165	7.400	[216.871, 227.458]	2.383
	1.0	197.535	3.307	[195.170, 199.901]	1.197

**Table 4:** Performance results for the simulation with swarms of different sizes.

ρ	IP (m)	AP (m)	*E* (m)	E (%)	TT (s)	OT (s)	ES
6	4.3284	4.1505	0.1778	4.1097	70.8	215.07	2.67
7	4.5322	4.3427	0.1894	4.1789	71.4	239.35	1.45
8	3.9142	3.7615	0.1526	3.9003	72.6	244.79	2.16

**Table 5:** Performance results for the simulation with swarms of 8 robots, wherein the object to be transported has different characteristics.

$r_{⊕}$ (m)	*M* (kg)	IP (m)	AP (m)	*E* (m)	*E* (%)	TT (s)	OT (s)	ES
0.30	0.3	3.9142	3.7615	0.1526	3.9003	72.6	244.79	3.91
0.40	0.7	4.3284	4.1598	0.1686	3.8954	75.0	282.47	1.38
0.45	1.0	4.3284	4.1809	0.1474	3.4073	73.2	258.62	2.49

**Table 6:** Performance results for the simulations for arenas with different discretization steps considering swarms of different sizes.

ρ	Step (m)	IP (m)	AP (m)	*E* (m)	E (%)	TT (s)	OT (s)	ES
6	0.5	4.5322	4.2901	0.2420	5.3396	121.2	280.11	4.90
	1.0	4.3284	4.1505	0.1778	4.1097	70.8	215.07	5.51
7	0.5	4.3284	4.0468	0.2815	6.5035	120.6	318.33	4.33
	1.0	4.5322	4.3427	0.1894	4.1789	71.4	239.35	5.56
8	0.5	4.7393	4.4172	0.3220	6.7942	127.8	330.00	4.46
	1.0	3.9142	3.7615	0.1526	3.9003	72.6	244.79	5.28

**Table 7:** Performance results for the simulations for arenas with different discretization steps considering a swarm of 8 robots and varying the object characteristics.

$r_{⊕}$ (m)	*M* (kg)	Step (m)	IP (m)	AP (m)	*E* (m)	E (%)	TT (s)	OT (s)	ES
0.30	0.3	0.5	4.7393	4.4172	0.3220	6.7942	127.8	330.00	1.59
		1.0	3.9142	3.7615	0.1526	3.9003	72.6	244.79	4.18
0.40	0.7	0.5	4.7393	4.4011	0.3381	7.1339	124.8	350.13	1.43
		1.0	4.3284	4.1598	0.1686	3.8954	75.0	282.47	2.88
0.45	1.0	0.5	4.3284	4.1128	0.2156	4.9810	121.2	312.09	2.04
		1.0	4.3284	4.1809	0.1474	3.4073	73.2	258.62	3.95

**Table 8:** Statistical results of the simulations for arenas with different discretization steps, considering swarms of different sizes, regarding the transport and overall times.

Step (m)	ρ	${\mathrm{AV}}_{\mathrm{TT}}$ (s)	$\sigma_{\mathrm{TT}}$	${\mathrm{CI}}_{\mathrm{TT}}$	${\mathrm{RE}}_{\mathrm{TT}}$ (%)	${\mathrm{AV}}_{\mathrm{OT}}$ (s)	$\sigma_{\mathrm{OT}}$	${\mathrm{CI}}_{\mathrm{OT}}$	${\mathrm{RE}}_{\mathrm{OT}}$ (%)
0.5	6	123.925	2.576	[122.082, 125.768]	1.487	287.849	3.625	[285.256, 290.442]	0.901
	7	129.051	6.823	[124.170, 133.932]	3.782	306.623	7.851	[301.007, 312.239]	1.831
	8	130.271	7.001	[125.263, 135.279]	3.844	322.716	6.970	[317.730, 327.702]	1.545
1.0	6	74.630	3.145	[72.380, 76.880]	3.014	209.018	12.257	[200.250, 217.786]	4.195
	7	78.739	9.270	[72.600, 79.078]	4.270	235.218	3.672	[232.591, 237.844]	1.116
	8	78.325	4.412	[75.169, 81.481]	4.029	250.097	7.435	[244.779, 255.415]	2.126

**Table 9:** Statistical results of the simulations for arenas with different discretization steps, considering swarms of different sizes, regarding the transport path error and efficiency scores.

Step (m)	ρ	${\mathrm{AV}}_{E}$ (m)	$\sigma_{E}$	${\mathrm{CI}}_{E}$	${\mathrm{RE}}_{E}$ (%)	${\mathrm{AV}}_{\mathrm{ES}}$ (m)	$\sigma_{\mathrm{ES}}$	${\mathrm{CI}}_{\mathrm{ES}}$	${\mathrm{RE}}_{\mathrm{ES}}$ (%)
0.5	6	0.246	0.0147	[0.236, 0.257]	4.267	5.022	0.2820	[4.820, 5.224]	4.017
	7	0.264	0.0146	[0.253, 0.274]	3.965	4.998	0.3440	[4.752, 5.244]	4.923
	8	0.316	0.0134	[0.307, 0.326]	3.036	5.142	0.2381	[4.972, 5.312]	3.312
1.0	6	0.169	0.0111	[0.161, 0.177]	4.690	5.500	0.1820	[5.369, 5.630]	2.367
	7	0.205	0.0630	[0.175, 0.184]	2.532	5.998	0.2669	[5.807, 6.189]	3.183
	8	0.206	0.0377	[0.158, 0.174]	4.897	5.229	0.311	[5.006, 5.451]	4.254

**Table 10:** Parameters for the compared simulations: pushing vs. caging.

Strategy	ρ	Arena ($m^{2}$)	#WP	*M* (kg)	$r_{⊕}$ (m)	Platform
Pushing ([26])	7	6.4×3.2	27	0.18	0.3	Robotarium Sim
Caging (proposed)	7	5.0×5.0	25	0.3	0.3	CoppeliaSim

**Table 11:** Average results for the simulation with swarms of 7 robots: pushing vs. caging.

Strategy	Step	Search	IP (m)	AP (m)	*E* (m)	*E* (%)	TT (s)	OT (s)	ES
Pushing ([26])	–	Rand	6.43	6.339	0.031	0.482	608.22	698.56	7.84
	–	Opt	6.38	6.363	0.017	0.263	608.22	717.10	9.67
Caging (proposed)	0.5	Rand	4.33	4.047	0.282	6.504	120.60	318.33	4.37
	1.0		4.53	4.343	0.190	4.179	71.40	239.35	17.42

## Data Availability

The original contributions presented in this study are included in the article. Further inquiries can be directed to the corresponding author.
